# Effectiveness of biomolecule-based bioactive surfaces, on os-seointegration of titanium dental implants: A systematic review and meta-analysis of *in vivo* studies

**DOI:** 10.3389/fbioe.2022.986112

**Published:** 2022-09-26

**Authors:** Nansi López-Valverde, Javier Aragoneses, Antonio López-Valverde, Norberto Quispe-López, Cinthia Rodríguez, Juan Manuel Aragoneses

**Affiliations:** ^1^ Department of Medicine and Medical Specialties, Faculty of Health Sciences, Universidad Alcalá de Henares, Madrid, Spain; ^2^ Department of Surgery, Instituto de Investigación Biomédica de Salamanca (IBSAL), University of Salamanca, Salamanca, Spain; ^3^ Department of Dentistry, Universidad Federico Henríquez y Carvajal, Santo Domingo, Dominican Republic; ^4^ Faculty of Dentistry, Universidad Alfonso X El Sabio, Madrid, Spain

**Keywords:** titanium dental implants, bioactive surface modifications, biomolecules, peptides, bone morphogenetic protein, grown factor, components of the extracellular matrix, osteointegration

## Abstract

Titanium and alloy osseointegrated implants are used to replace missing teeth; however, some fail and are removed. Modifications of the implant surface with biologically active substances have been proposed. MEDLINE [via Pubmed], Embase and Web of Science were searched with the terms “titanium dental implants”, “surface properties”, “bioactive surface modifications”, “biomolecules”, “BMP”, “antibacterial agent”, “peptide”, “collagen”, “grown factor”, “osseointegration”, “bone apposition”, “osteogenic”, “osteogenesis”, “new bone formation”, “bone to implant contact”, “bone regeneration” and “*in vivo* studies”, until May 2022. A total of 10,697 references were iden-tified and 26 were included to analyze 1,109 implants, with follow-ups from 2 to 84 weeks. The ARRIVE guidelines and the SYRCLE tool were used to evaluate the methodology and scientific evidence. A meta-analysis was performed (RevMan 2020 software, Cochane Collaboration) with random effects that evaluated BIC at 4 weeks, with subgroups for the different coatings. The heterogeneity of the pooled studies was very high (95% CI, I2 = 99%). The subgroup of BMPs was the most favorable to coating. Surface modification of Ti implants by organic bioactive molecules seems to favor osseointegration in the early stages of healing, but long-term studies are necessary to corroborate the results of the experimental studies.

## 1 Introduction

Since the introduction of dental implants by Brånemark in the 1960s, titanium (Ti) and some Ti alloys (Ti6Al4V) have been used in edentulous patients to replace missing teeth (Osman and Swain, 2015), their long-term success depending mainly on their osseointegration. However, despite the high success rates recorded, some of them have to be removed due to failure (Moraschini et al., 2015; Alghamdi and Jansen, 2020).

Recently, the attention of researchers has been focused on chemical and topographical modifications of dental implant surfaces and surface coatings with biologically active materials (Le Guéhennec et al., 2007).

These materials, in addition to provoking a response in living tissue, would have the capacity to achieve a faster, higher quality and more durable osseointegration, reducing the waiting time for prosthetic rehabilitations and solving the problems of poor bone quality (Stanford, 2008). Currently, bioceramics, ions and biomolecules are applied for bioactive purposes (Ellingsen et al., 2004; Cooper et al., 2006; Badr and Hadary, 2007; Zagury et al., 2007). The latter include biomacromolecules (lipids, proteins, polynucleic acids and polysaccharides) and biomicromolecules (oligopeptides, deoxyribonucleotides, amino acids, monosaccharides and metabolic products), which are of extraordinary importance for physiological processes and homeostasis (Fischer et al., 2020).

The ability to adhere to bone tissue and the chemical similarity with this tissue have led to great interest in calcium phosphate (CaP) coatings on the surface of implants, precisely because they increase the biochemical anchorage between the bone and the surface materials (Bosco et al., 2013). Similarly, protein coatings have been used in recent years because they accelerate the bone regeneration process at the bone-implant interface and improve osseointegration (Raphel et al., 2016).

Bone morphogenetic protein (BMP) and collagen have been proposed as bone regeneration stimulating materials. Collagen is an important component in bone composition, leading to increased tissue vascularization and decreased inflammation by curbing macrophage and osteoclast activity (Lee et al., 2014). In turn, BMPs play an important role in osteogenesis by regulating the differentiation of bone mesenchymal stem cells (MSCs) and osteogenic cells (Dolanmaz et al., 2015a).

Synthetic peptides have been shown to stimulate bone formation by enhancing the binding of osteoblast cell adhesion receptors (e.g., integrins, selectins, and cadherins). Binding of osteoblast integrin receptors to these bioactive molecules stimulates their interaction with their extracellular matrix (ECM) and promotes cell proliferation and mineralization (Garcia and Reyes, 2005).

Studies have shown that biofunctionalization of implant surfaces with biomimetic peptides would result in a greater increase in the bone-to-implant contact surface (BIC) and an increase in bone density around the implant (Lutz et al., 2010a). However, the process of peptide immobilization on Ti implant surfaces can be a complex process, despite the fact that, in recent years, specific methods have been developed to achieve this goal (Narai and Nagahata, 2003; Russell et al., 2008; Viera-Negron et al., 2008). Also, it has been observed that the biological activity of certain peptides would be reduced by the immobilization process. The surface density, together with the length of the spacers and the orientation, would condition the bactericidal effect of the peptides (Giro et al., 2008). Moussa and Aparicio demonstrated *in vitro* that bacterial abundance on peptide-coated hydroxyapatite (HA) discs was significantly lower than in controls (Andrea et al., 2018a). Makihira et al. tested in edentulous dog mandibles, the osseointegrative capacity of Ti implants coated with a histatin-derived peptide, demonstrating, by histological and micro-CT analysis, increased trabecular bone formation around the coated implants (Riool et al., 2017). Their observations suggest that antimicrobial peptides on Ti implants would decrease bacterial colonization on the implant surface and facilitate osseointegration (Silva et al., 2016; Zhang et al., 2018).

Despite the existence in the literature of reviews to evaluate the effects of different implant surface modifications on peri-implant bone formation and osseointegration (Makihira et al., 2011; Andrea et al., 2018b; Moussa and Aparicio, 2020; Siwakul et al., 2021) and the known benefit on osseointegration of the use of bioactive molecules (Junker et al., 2009), we have not found meta-analyses that investigate the results in depth, so the aim of our study was to evaluate the role and efficacy of bioactive surfaces on osseointegration. Our meta-analysis limited the research interest to titanium dental implants coated with biomolecules, i.e. organic molecules produced by a living organism.

## 2 Materials and methods

### 2.1 Registration

This systematic review was registered at INPLASY, registration number INPLASY202260076.

### 2.2 PICOS and focused question

Supplementary Table S1: PRISMA Checklist]. According to the PRISMA guidelines for Systematic Reviews and Meta-Analyses (Hutton et al., 2016), a specific question was formulated based on the PICOS principle (Participants, Interventions, Control, Outcomes, and Study Design). The focused question was, “Does the bioactive surface of titanium dental implants, based on biomolecules, influence osseointegration?“.

P) Participants: Subjects received endosseous implantation.
I) Interventions: Implants with incorporated bioactive surfaces based on biomolecules.
C) Control: Implants with conventional etched surfaces (SLA type).
O) Outcome: Bone to Implant Contact (BIC).
S) Study design: Preclinical studies in unmodified experimental animal models.

### 2.3 Search strategy

The electronic databases PubMed/MEDLINE, WOS and EMBASE were searched until May 2022, with the terms Medical Subject Headings (MeSH): “titanium dental implants”, “surface properties”, “bioactive surface modifications”, “biomolecules”, “BMP”, “antibacterial agent”, “peptide”, “collagen”, “grown factor”, in combination with “osseointegration”, “bone apposition”, “osteogenic”, “osteogenesis”, “new bone formation”, “bone to implant contact”, “bone regeneration” and “*in vivo* studies”. The Boolean operators AND/OR were used to refine the search. In addition, relevant studies in the gray literature and reference lists of included studies were also examined (cross-referenced). The search strategy and the PICOS strategy are shown in Table 1.

**TABLE 1:** Systematic search strategy (PICOS strategy).

Population	Experimental animals receiving implants with bioactive surfaces based on biomolecules
Intervention	Intraosseous implant treatments
Comparisons	Intraosseous implants with conventional etched surfaces (SLA type)
Outcomes	Bone to Implant contact (BIC)
Study design	Preclinical studies in unmodified experimental animal models
Search combination	#1 AND #2 OR
Language	English
Electronic databases	PubMed/MEDLINE, WOS and EMBASE

### 2.4 Inclusion and exclusion criteria

#### 2.4.1 Inclusion criteria

1) Studies regarding Ti implant surfaces coated with biomolecules; b) Studies reporting evaluation of the effect of biomolecular coatings on bone formation or osseointegration; 3) Studies published in English.

#### 2.4.2 Exclusion criteria

1) *In vitro* studies; b) Studies using modified animals; 3) Narrative reviews and systematic reviews; 4) Irrelevant and duplicate studies and those that did not meet the established inclusion criteria.

### 2.5 Data extraction and analysis

Studies that did not refer to the research question were eliminated and only the titles and abstracts of the selected articles were considered and entered into an Excel spreadsheet. Two reviewers (N.L.-V. and A.L.-V.) selected the titles and abstracts independently. Discrepancies between the two reviewers were discussed until a consensus was reached for inclusion of the studies. The full texts of the selected studies were then obtained for inclusion and analysis.

### 2.6 Risk of bias of included articles

An adapted version of the Cochrane RoB tool with specific biases in animal studies (SYRCLE) was used to assess the scientific evidence in all selected studies (Hooijmans et al., 2014).

### 2.7 Quality of the reports of the included studies

Two reviewers N.L.-V. and A.L.-V evaluated the included studies according to the ARRIVE (Animal Research: Reporting of *In Vivo* Experiments) guidelines (Stadlinger et al., 2012a), which include a total of 23 items. Each item was scored by 0 (not reported) or 1 (reported), with a complete count of all included studies.

### 2.8 Statistical analysis

The meta-analysis was performed using RevMan software [Review Manager (RevMan) (Computer program). Version 5.4.1, The Cochrane Collaboration, 2020].

A meta-analysis based on Odds Ratio (OR) with 95% confidence intervals (CI) was performed for adverse event outcomes. Mean difference (MD) and standard deviation (SD) were used to estimate effect size. The random-effects model was selected because of the expected methodological heterogeneity in the included studies; furthermore, heterogeneity was interpreted as significant when the I2 value was >50%. The threshold for statistical significance was defined as *p* < 0.05. A funnel plot was used to assess publication bias.

## 3 Results

### 3.1 Selection and description of the studies

**FIGURE 1:**
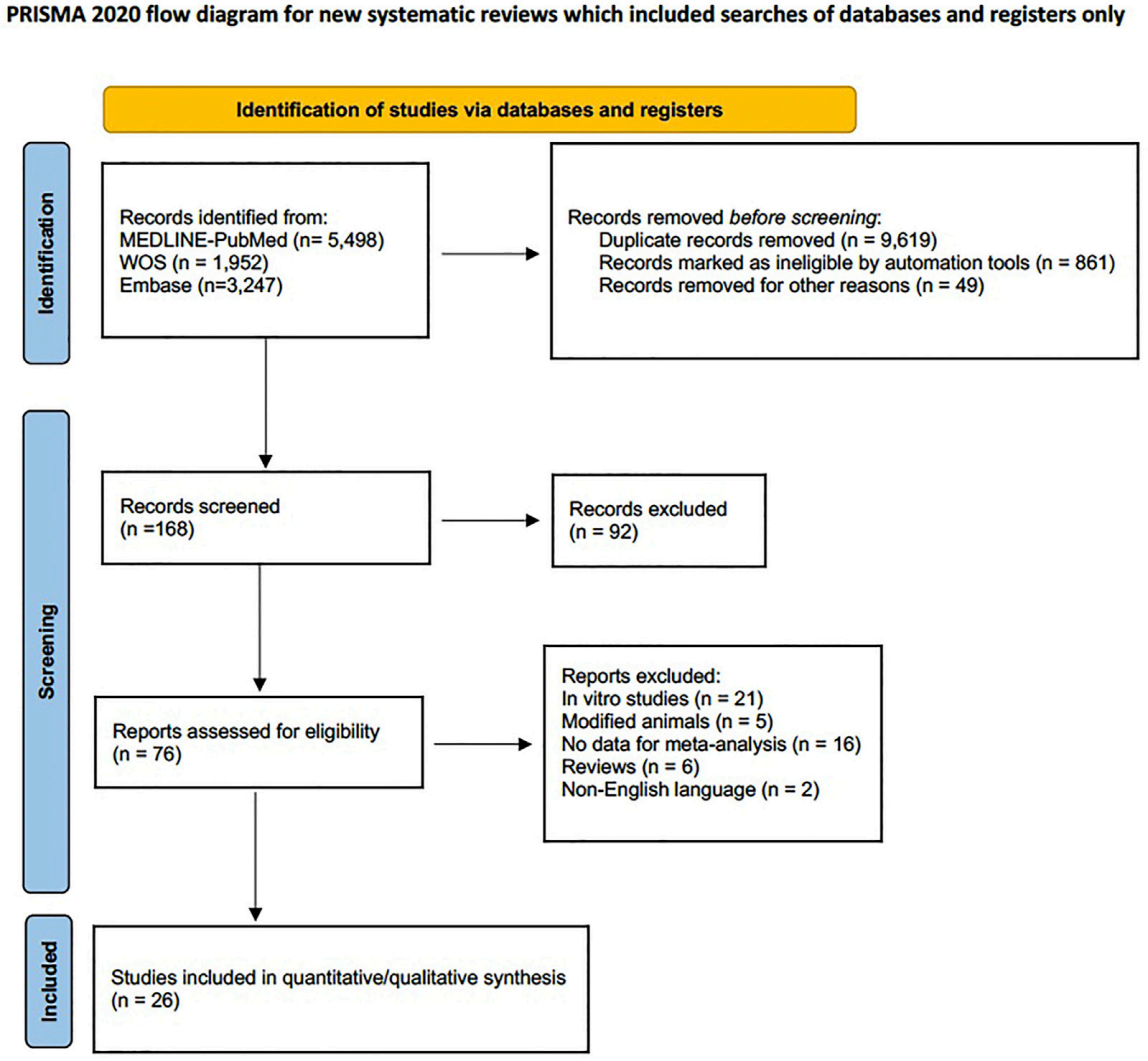
Flowchart.

Among the available literature, three categories of biomolecular coatings have been evaluated in this review: 1) peptides, 2) BMPs and 3) ECM. The initial electronic search yielded 10,697 references. After eliminating duplicates and irrelevant articles based on their title and abstracts, 84 articles were selected, of which, after eliminating those that did not meet inclusion criteria (*in vitro* studies, systematic reviews, modified animals...), 26 full texts were selected (Anitua, 2006; Germanier et al., 2006; Wikesjö et al., 2008a; Wikesjö et al., 2008b; Wikesjö et al., 2008c; Stadlinger et al., 2008; Anitua et al., 2009; Barros et al., 2009; Ishibe et al., 2009; Yang et al., 2009; Lutz et al., 2010b; Polimeni et al., 2010; Susin et al., 2010; Ramazanoglu et al., 2011; Stadlinger et al., 2012b; Sverzut et al., 2012; Jiang et al., 2013; Cecconi et al., 2014; Korn et al., 2014; Kim et al., 2015; Yoo et al., 2015; Cardoso et al., 2017; Bae et al., 2018; Cho et al., 2019; Cho et al., 2021; Pang et al., 2021). The concordance between reviewers (N.L-V., A.L-V.) was 100% with a Cohen’s kappa index of 1 (total concordance). (Figure 1. Flow Diagram).

Table 2 provides the evaluation of the ARRIVE criteria in animal studies, with a mean rating of 16.5 ± 1.5. All studies provided adequate information in terms of title, abstract, introduction, ethical statement, species, surgical procedure, outcome assessment and statistical analysis. Items 5 (Rationale for animal models), 19 (3Rs, Replace, Reduce and Refine), 20 (Adverse events), were not reported in any of the included studies. Item 11 (Accommodation and handling of animals) was reported by only five studies (Anitua, 2006; Anitua et al., 2009; Ishibe et al., 2009; Lutz et al., 2010b; Korn et al., 2014) and item 21 (Study limitations) was reported by six studies (Jiang et al., 2013; Korn et al., 2014; Kim et al., 2015; Yoo et al., 2015; Cardoso et al., 2017; Bae et al., 2018).

**TABLE 2:** Checklist of ARRIVE criteria reported by the included studies. Each item was judged as “0” (not reported) or “1” (reported).

Studies	Germanier et al., 2006 (Germanier et al., 2006)	Anitua 2006 (Anitua, 2006)	(1) wikesjö et al., 2008 (Wikesjö et al., 2008a)	(2) wikesjö et al., 2008 (Wikesjö et al., 2008b)	(3) wikesjö et al., 2008 (Wikesjö et al., 2008c)	Stadlinger et al., 2008 (Stadlinger et al., 2008)	Barros et al., 2009 (Barros et al., 2009)	Yang et al., 2009 (Yang et al., 2009)	Anitua et al., 2009 (Anitua et al., 2009)	Ishibe et al., 2009 (Ishibe et al., 2009)	Lutz et al., 2010 (Lutz et al., 2010b)	Susin et al., 2010 (Susin et al., 2010)	Polimeni et al., 2010 (Polimeni et al., 2010)
1 Title	1	1	1	1	1	1	1	1	1	1	1	1	1
Abstract													
2 Species	1	1	1	1	1	1	1	1	1	1	1	1	1
3 Key finding	1	1	1	1	1	1	1	1	1	1	1	1	1
Introduction													
4 Background	1	1	1	1	1	1	1	1	1	1	1	1	1
5 Reasons for animal models	0	0	0	0	0	0	0	0	0	0	0	0	0
6 Objectives	1	1	1	1	1	1	1	1	1	1	1	1	1
Methods													
7 Ethical statement	1	1	1	1	1	1	0	1	1	1	1	1	1
8 Study design	1	1	1	1	1	1	1	1	1	1	1	1	1
9 Experimental procedures	1	1	1	1	1	1	1	1	1	1	1	1	1
10 Experimental animals	1	1	1	1	1	1	1	1	1	1	1	1	1
11 Accommodation and handling of animals	0	1	0	0	0	0	0	0	1	1	1	0	0
12 Sample size	1	1	1	1	1	1	1	1	1	1	1	1	1
13 Assignment of animals to experimental groups	1	1	1	1	1	1	0	0	1	1	1	1	1
14 Anaesthesia	1	1	1	1	1	1	1	1	1	1	1	1	1
15 Stadistical methods	1	1	1	1	1	1	1	1	1	1	1	1	1
Results													
16 Experimental results	1	1	1	1	1	1	1	1	1	1	1	1	1
17 Results and estimation	0	1	0	1	1	1	1	1	1	1	1	1	1
Discussion													
18 Interpretation and scientific implications	1	1	0	1	0	1	0	1	1	1	0	0	0
19 3Rs reported	0	0	0	0	0	0	0	0	0	0	0	0	0
20 Adverse events	0	0	0	0	0	0	0	0	0	0	0	0	0
21 Study limitations	0	0	0	0	0	0	0	0	0	0	0	0	0
22 Generalization/applicability	0	1	0	0	0	1	0	0	1	0	0	0	0
23 Funding	0	0	1	1	1	1	1	0	0	0	1	1	1
TOTAL SCORE	15	18	15	17	16	18	14	15	18	17	17	16	16

Studies	Ramazanoglu et al., 2011 (Ramazanoglu et al., 2011)	Stadlinger et al., 2012 (Stadlinger et al., 2012b)	Sverzut al. 2012 (Sverzut et al., 2012)	Jiang et al., 2013 (Jiang et al., 2013)	Cecconi et al., 2014 (Cecconi et al., 2014)	Korn et al., 2014 (Korn et al., 2014)	Kim et al., 2015 (Kim et al., 2015)	Yoo et al., 2015 (Yoo et al., 2015)	Cardoso et al., 2017 (Cardoso et al., 2017)	Bae et al., 2018 (Bae et al., 2018)	Cho et al., 2019 (Cho et al., 2019)	Pang et al., 2021 (Pang et al., 2021)	Cho et al., 2021 (Cho et al., 2021)
1. Title	1	1	1	1	1	1	1	1	1	1	1	1	1
Abstract													
2. Species	1	1	1	1	1	1	1	1	1	1	1	1	1
3. Key finding	1	1	1	1	1	1	1	1	1	1	1	1	1
Introduction													
4. Background	1	1	1	1	1	1	1	1	1	1	1	1	1
5. Reasons for animal models	0	0	0	0	0	0	0	0	0	0	0	0	0
6. Objectives	1	1	1	1	1	1	1	1	1	1	1	1	1
Methods													
7. Ethical statement	1	1	1	1	1	1	1	1	1	1	1	1	1
8. Study design	1	1	1	1	1	1	1	1	1	1	1	1	1
9. Experimental procedures	1	1	1	1	1	1	1	1	1	1	1	1	1
10. Experimental animals	1	1	1	1	1	1	1	1	1	1	1	1	1
11. Accommodation and handling of animals	0	0	0	0	0	1	0	0	1	0	0	0	0
12. Sample size	1	1	1	1	1	1	1	1	1	1	1	1	1
13. Assignment of animals to experimental groups	1	1	0	1	0	1	1	1	1	1	0	0	1
14. Anaesthesia	1	1	1	1	1	1	1	1	1	1	1	0	1
15. Stadistical methods	1	1	1	1	1	1	1	1	1	1	1	1	1
Results													
16. Experimental results	1	1	1	1	1	1	1	1	1	1	1	1	1
17. Results and estimation	1	1	1	1	1	1	1	1	1	1	1
Discussion												
18. Interpretation and scientific implications	1	0	0	1	0	1	1	0	1	0	0	0	1
19. 3Rs reported	0	0	0	0	0	0	0	0	0	0	0	0	0
20. Adverse events	0	0	0	0	0	0	0	0	0	0	0	0	0
21. Study limitations	0	0	0	1	0	1	1	1	1	1	0	0	1
22. Generalization/applicability	0	0	0	0	0	0	0	0	0	0	1	1	0
23. Funding	1	0	1	1	1	1	1	1	1	1	1	0	1
TOTAL SCORE	17	15	15	18	15	19	18	18	19	17	16	13	17

Mean rating: 16.5 ± 1.5.

### 3.2 Risk of bias assessment

**FIGURE 2:**
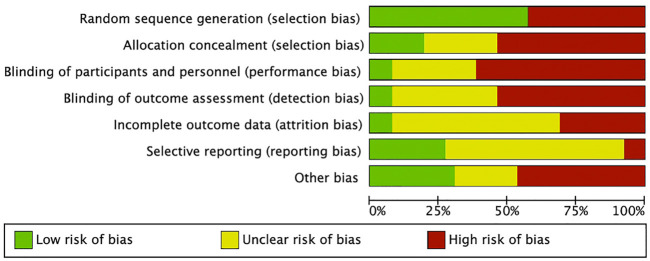
SYRCLE’s risk of bias tool.

The Random sequence generation domain was the most frequently mentioned (60%). Blinding of participants and personnel and Blinding of outcome assessment were the least mentioned domains. The domains Incomplete outcome data and Selective reporting were the least clear. The lack of information resulted in a high and unclear risk of bias for most of the included studies (Figure 2).

### 3.3 Characteristics of the included studies

Qualitative synthesis. A total of 1,109 implants were evaluated. Most of the studies employed commercial Ti and Ti alloy implant models, with the exception of two studies in rat tibias (Ishibe et al., 2009; Bae et al., 2018) that used rods and microscrews, respectively. The implants featured either a re-coated or uncoated surface with peptides in five studies (Germanier et al., 2006; Barros et al., 2009; Yang et al., 2009; Lutz et al., 2010b; Cho et al., 2019), BMPs in fourteen studies (Anitua, 2006; Wikesjö et al., 2008a; Wikesjö et al., 2008b; Wikesjö et al., 2008c; Anitua et al., 2009; Ishibe et al., 2009; Polimeni et al., 2010; Susin et al., 2010; Ramazanoglu et al., 2011; Kim et al., 2015; Yoo et al., 2015; Cardoso et al., 2017; Pang et al., 2021), or ECM products in seven studies (Stadlinger et al., 2008; Stadlinger et al., 2012b; Sverzut et al., 2012; Cecconi et al., 2014; Korn et al., 2014; Bae et al., 2018; Cho et al., 2019). Follow-up periods ranged from 2 to 16 weeks, except for the study by Bae et al. (Bae et al., 2018) that the follow-up period was extended to 84 weeks. The most commonly used experimental models were the dog (Wikesjö et al., 2008a; Wikesjö et al., 2008b; Barros et al., 2009; Polimeni et al., 2010; Susin et al., 2010; Sverzut et al., 2012; Kim et al., 2015; Cho et al., 2019) and the pig (Germanier et al., 2006; Stadlinger et al., 2008; Lutz et al., 2010b; Susin et al., 2010; Ramazanoglu et al., 2011; Stadlinger et al., 2012b; Korn et al., 2014; Cardoso et al., 2017). The jaw and tibia were the most commonly used bones for implantation and all included studies evaluated the BIC; six studies evaluated BA (Yang et al., 2009; Sverzut et al., 2012; Yoo et al., 2015; Cho et al., 2019; Cho et al., 2021; Pang et al., 2021) and nine studies evaluated BD (Wikesjö et al., 2008b; Wikesjö et al., 2008c; Stadlinger et al., 2008; Barros et al., 2009; Lutz et al., 2010b; Polimeni et al., 2010; Susin et al., 2010; Ramazanoglu et al., 2011; Korn et al., 2014). The main characteristics of the studies are shown in the tables below (Tables 3–5).

**TABLE 3:** Surface modification with peptides. Characteristics of the included studies.

Study	Animal model	Biomolecule	Implantation site	Length of study	Implanted device (length and diameter mm)	Material and number of implanted devices	Parameters measured	Findings
Cho et al., 2019 (Cho et al., 2019)	Rabbit	A human vitronectin-derived peptide	Tibiae	2 weeks	11 × 3.5 Ø	Ti, grade 4 (16)	BIC, BA	There were no significant differences in BIC and BA between the groups
Germanier et al. (Germanier et al., 2006)	Pig	RGD-peptide-modified polymer	Maxilla	2 and 4 weeks	6 × 2.7 Ø	Commercially pure Ti (48)	BIC	Bone tissue scaffolding was observed at 2 weeks, increasing bone density at 4 weeks
Lutz et al. (Lutz et al., 2010b)	Pig	Biomimetic active peptide (P-15)	Forehead region	2 and 4 weeks	8 × 3.5 Ø	Commercially pure Ti (54)	BIC, BD	Significant positive effect of the biomimetic peptide group on BIC with high contact rates at both 14 and 30 days. The biomimetic peptide had no significant effect on peri-implant BD
Barros et al. (Barros et al., 2009)	Dog	Bioactive peptide (sequence of aminoacids related to bone formation)	Mandible	8 weeks	9.5 × 4.5 Ø	Commercially pure Ti (48)	BIC, BD	Bone apposition and bone density around Ti implants depended on bioactive peptide concentrations
Yang et al. (Yang et al., 2009)	Rabbit	RGD layer-by-layer	Femur	4, 8, and 12 weeks	10 × 3 Ø	Ti (60)	BIC, BA, RTQ	RGD coating results in increased BIC, peri-implant bone formation and extraction torque values

Ti, Titanium; BIC, bone to implant contact; BA, bone area; BD, bone density; RTQ, removal torque test; RGD, Arginine-glycine-aspartic.

**TABLE 4:** Surface modification with Bone Morphogenetic Proteins (BMPs). Characteristics of the included studies.

Study	Animal model	Biomolecule	Implantation site	Length of study	Implanted device (length and diameter mm)	Material and number of implanted devices	Parameters measured	Findings
Kim et al. (Kim et al., 2015)	Dog	rhBMP-2	Tibiae	8 weeks	7 × 3.5 Ø	Pure Ti (24)	BIC, BV, ISQ	Concentrations of 0.5 and 1 mg/ml rhBMP-2 promote osseointegration and bone regeneration in areas with open bone defects
Pang et al. (Pang et al., 2021)	Rabbit	BMP-2+HA	Tibiae	4 weeks	7 × 3.3 Ø	Pure Ti (8)	BIC, BA, RTQ	The combination of BMP-2 with HAp functions as an activator of osseointegration
Yoo et al. (Yoo et al., 2015)	Rabbit	rhBMP-2/PLGA	Tibiae	3 and 7 weeks	7 × 3.75 Ø	Pure grade IV Ti (32)	BIC, BA	Submicron-sized PLGA/rhBMP-2 Ti coatings showed an increase in BIC during the early stages of healing
Cardoso et al. (Cardoso et al., 2017)	Pig	PPL10BMP	Parietal bone	4, 8 and weeks	6 × 1.1 Ø	Pure Ti (120)	B/T, BIC	The association of PPL10 and BMP-2 did not produce a bone improvement
Ishibe et al. (Ishibe et al., 2009)	Rat	rhBMP-2/heparin	Tibiae	3 weeks	2 × 1 Ø	Pure Ti (70)	BIC	The incorporation of BMP-2 and heparin has the potential to stimulate new bone formation around implants *in vivo*
Jiang et al. (Jiang et al., 2013)	Rabbit	rhBMP-2	Femur	2, 4 and 8 weeks	8 × 4.1 Ø	Pure Ti (30)	BIC	Acid-etched titanium implants coated with BMP-2 slightly accelerated early bone formation around the implant
Susin et al. (Susin et al., 2010)	Dog	rhBMP-7	Jaw	3, 4, 7, and 8 weeks	10 × 4 Ø	Ti (36)	BIC, BD	Porous titanium oxide implants coated with rhBMP-7 stimulated bone formation and osseointegration
Polimeni et al. (Polimeni et al., 2010)	Dog	rhGDF-5	Jaw	3, 4, 7, and 8 weeks	10 × 4 Ø	Ti (72)	BIC, BD	Dental implants coated with rhGDF-5 showed a dose-dependent osteoinductive and/or osteoconductive effect
Ramazanoglu et al. (Ramazanoglu et al., 2011)	Pig	rhBMP-2+rhVEGF1_65_	Calvaria	1, 2, and 4 weeks	6 × 4.2 Ø	Pure Ti (90)	BIC, BD, BV	The combined administration of rhBMP-2 and rhVEGF165 in biomimetic coating did not result in an improvement of BIC
Wikesjö et al. (Wikesjö et al., 2008a) (1)	Dog	rhBMP-2 (0,75 or 1.5 mg/ml)	Jaw	3, 4, 7 and 8 weeks	10 × 4 Ø	Ti (72)	BIC, BD	The implant surfaces coated with rhBMP-2 induced osseointegration, but BIC values were significantly higher in the control group
Wikesjö et al. (Wikesjö et al., 2008b) (2)	Dog	rhBMP-2 (0.2 or 4.0 mg/ml)	Jaw	4 and 8 weeks	8.5 × 3,75Ø	Ti (32)	BIC, BD	Adsorbed rhBMP-2 on implant surfaces initiates dose-dependent peri-implant bone remodelling
Wikesjö et al. (Wikesjö et al., 2008c) (3)	Monkey	rhBMP-2 (0,2 or 2 mg/ml)	Maxilla	16 weeks	8.5 × 3,75Ø	Ti (24)	BIC, BD	The rhBMP-2 coated Ti surface enhances/accelerates local bone formation in type IV bone resulting in significant osseointegration
Anitua (Anitua, 2006) (1)	Goat	PRGF	Tibiae and radii	8 weeks	8.5 x 3Ø	Ti (23)	BIC	Coating dental implants with PRGF immediately before insertion improved osseointegration
Anitua (Anitua et al., 2009) (2)	Goat	PRGF	Tibiae	8 weeks	8.5 x 3Ø	Ti (26)	BIC	Hydration of titanium implants with liquid PRGF improves the integration of oral implants into cortical bone. The potential therapeutic effects of this approach could be extrapolated to other prosthetic devices

Ti, Titanium; BIC, bone to implant contact; BV, bone volume; BA, bone area; ISQ, implant stability quotient; HA, hydroxyapatite; PLGA, poly(d,l-lactide-co-glycolide); PPL10, 10% phosphorylated pullulan; Peri-implant bone formation (B/T); BD, bone density; rhGDF-5, recombinant human GDF-5; rhVEGF1_65,_ recombinant human vascular endothelial growth factor; rhBMP-2, recombinant human bone morphogenetic protein-2; PRGF, plasma rich in growth factors.

**TABLE 5:** Surface modification with ECM. Characteristics of the included studies.

Study	Animal model	Biomolecule	Implantation site	Length of study	Implanted device (length and diameter) mm	Material and number of implanted devices	Parameters measured	Findings
Sverzut et al. (Sverzut et al., 2012)	Dog	Type I Collagen	Jaw	3 weeks	8.5 × 3.75 Ø	Ti (24)	BIC, BA	The collagen coating of Ti implants improves osteoinduction and tissue vascularization while reducing inflammatory response and macrophage and osteoclast activity
Stadlinger et al. (Stadlinger et al., 2008) (1)	Pig	Type I Collagen/rhBMP-4	Jaw	3 and 7 weeks	12 × 4.25 Ø	Ti (120)	BIC	The inclusion of chondroitin sulfate in the coating increases the BIC of collagen-coated implants, however, the additional inclusion of a low amount of rhBMP-4 had a detrimental effect
Cho et al. (Cho et al., 2021)	Dog	Type I Collagen/GA	Jaw	8 weeks	8 × 4 Ø	Pure Ti (36)	BIC, BA	Gamma-irradiated collagen crosslinking is as effective as GA crosslinking in terms of bone regeneration efficiency
Bae et al. (Bae et al., 2018)	Rat	Type I Collagen/GA	Tibia	84 weeks	2.5 × 1.5 Ø	Ti (12)	BIC, NBV	Radiation cross-linked collagen-coated Ti implants possess potential osteoinductive qualities without the adverse effects of chemical agents
Korn et al. (Korn et al., 2014)	Pig	Collagen/CS/sHya	Jaw	4 and 8 weeks	15 × 5 Ø	Ti (36)	BIC, BD	Collagen/CS/sHya-coated Ti implants did not show an increase in BIC compared to the acid-etched and blasted References surface. However, they did increase bone density compared to the References surface
Stadlinger et al. (Stadlinger et al., 2012b) (2)	Pig	Collagen/CS	Jaw	4 and 8 weeks	9.5 × 4.5 Ø	Ti (120)	BIC, BD	The coatings did not show a significant effect on BIC or BVD.
Cecconi et al. (Cecconi et al., 2014)	Rabbit	Type I Collagen/Apatite	Femur	7 weeks	8.5 × 4 Ø	Ti (24)	BIC	Coating with bone apatite and type I collagen increased new bone formation and bone attachment around Ti implants

Ti, Titanium; BIC, bone to implant contact; BV, bone volume; BA, bone area; rhBMP-2, recombinant human bone morphogenetic protein-2; GA, glutaraldehyde; NBA, new bone area; ITBD, inter-thread bone densities; NBV, new bone volume; CS, chondroitin sulfate; BVD, bone volume density; sHya, sulfated hyaluronan.

### 3.4 Quantitative synthesis (meta-analysis)

**FIGURE 3:**
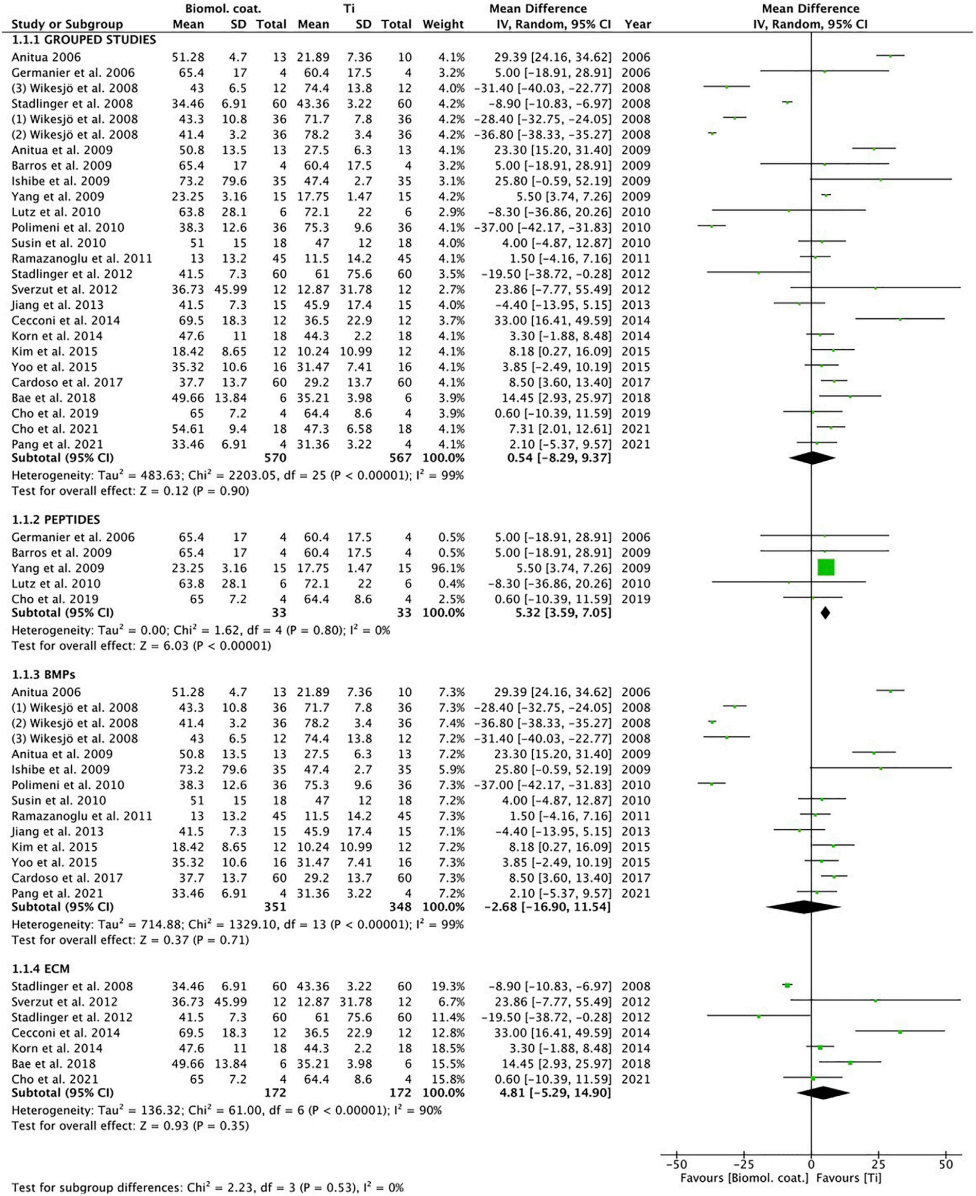
Forest plot for meta-analysis of studies evaluating BIC at 4 weeks after placement, assuming a random-effects model. SD, standard deviation; CI, confidence interval.

The same studies included in the qualitative synthesis were used to perform a meta-analysis comparing Ti implants coated with different biomolecules, with Ti implants etched. Meta-analysis of adverse outcomes could not be performed due to lack of data. All included studies (Anitua, 2006; Germanier et al., 2006; Wikesjö et al., 2008a; Wikesjö et al., 2008b; Wikesjö et al., 2008c; Stadlinger et al., 2008; Anitua et al., 2009; Barros et al., 2009; Ishibe et al., 2009; Yang et al., 2009; Lutz et al., 2010b; Polimeni et al., 2010; Susin et al., 2010; Ramazanoglu et al., 2011; Stadlinger et al., 2012b; Sverzut et al., 2012; Jiang et al., 2013; Cecconi et al., 2014; Korn et al., 2014; Kim et al., 2015; Yoo et al., 2015; Cardoso et al., 2017; Bae et al., 2018; Cho et al., 2019; Cho et al., 2021; Pang et al., 2021) evaluated bone-to-implant contact (BIC), using measurement 4 weeks after placement. The heterogeneity of the grouped studies was very high (I^2^ = 99%) (Figure 3). Only one result favorable to coating, was found in the BMPs subgroup. Analysis of the grouped studies showed no significant differences between coatings and controls.

### 3.5 Publication bias and heterogeneity

**FIGURE 4:**
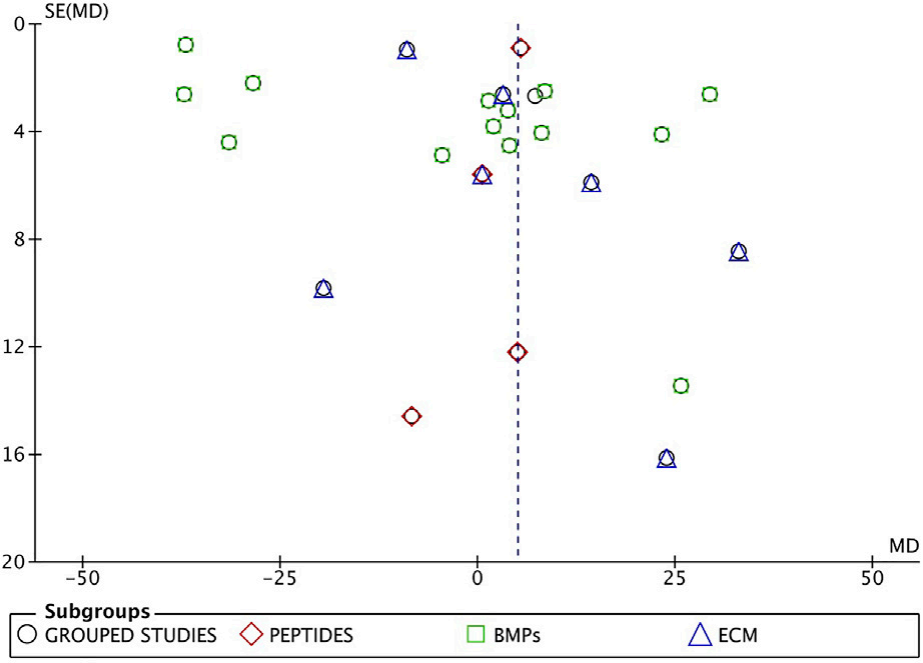
Funnel plot of grouped studies. The asymmetry proves publication bias.

The grouped studies show graphic signs of publication bias (Figure 4).

## 4 Discussion

The purpose of the present study was to answer the following clinical question: “Does the bioactive surface of titanium dental implants, based on biomolecules, influence osseointegration?”.

Osseointegration is the stable anchorage of an implant through direct bone-to-implant contact (Albrektsson and Johansson, 2001).

The main objective of surface modifications of endosseous implants is to modulate the response of the host bone tissue to achieve better osseointegration.

This review focused on BIC analysis in three categories of biomolecular Ti implant coatings: peptides, BMPs and ECM and identified 26 preclinical research articles that used BIC analysis to assess peri-implant bone formation in different animal models. The included studies found that coatings with bioactive molecules increased bone values around the implant; only the study by Ramazanoglu et al. (2011) found no difference in BIC in the rhBMP-2 coating.

After insertion of an endosseous implant, a series of events occur between the host and the implant surface. During the intercommunication of the implant surface and the blood of the recipient, ligands and proteins are dynamically adsorbed at the implant surface and through a subsequent inflammatory process are released from it, followed by bone formation around the bioactive surface, reaching the maximum degree of organization and biomechanical properties through several remodeling cycles (Lemons, 2004; Goiato et al., 2009). Due to the dynamic nature of the bone-biomaterial interface, biomaterials for endosseous dental implants must have short- and long-term biocompatible and biofunctional properties (Xuereb et al., 2015). It was Puleo and Nanci (Puleo and Nanci, 1999), in 1999, who first indicated that “biochemical surface modification strives to utilize current knowledge of the biology and biochemistry of cell function and differentiation".

Since then, and especially in recent years, surface modifications of Ti and Ti6Al4V implants, using methods based on the immobilization of biologically active organic molecules, have aroused particular interest among researchers, with the aim of increasing cell migration and adhesion to the substrate and avoiding nonspecific addition of proteins, to improve the healing process (Panayotov et al., 2015). (Drexelius and Neundorf, 2021) Antimicrobial peptides have evolved as reliable alternatives to commonly used antibiotics and are positioned as candidates for antimicrobial surface coatings of implants. A review by Drexelius and Neundorf concluded that they have excellent *in vitro* and *in vivo* antimicrobial activity (Drexelius and Neundorf, 2021). Kang et al. (Kang et al., 2013) in a mixed *in vitro* and *in vivo* study used a laminin-2-derived peptide capable of promoting initial cell adhesion and propagation of osteoblast-like cells *in vitro*, acting as an accelerator of osseointegration of implant materials and determining its positive effect, *in vivo*, on BIC values.

Plasma and extracellular matrix proteins (type I collagen, fibronectin, vitronectin, osteopontin, and bone sialoprotein), which contain at binding sites the RGD (Arg-Gly-Asp) sequence, together with receptor integrins, constitute an important recognition system for cell adhesion (Ruoslahti, 1996). Two of the selected studies (Germanier et al., 2006; Yang et al., 2009) investigated the effect of RGD coating by a layered self-assembly technique on porous surface implants, concluding that the peptides possess potential to transmit particular cell adhesion properties to Ti surfaces and are able to enhance cell-material interactions. Kroese-Deutman et al. (Kroese-Deutman et al., 2005) used a porous Ti fiber mesh implant coated with the RGD peptide in the rabbit skull and compared it with porous Ti fiber mesh disks without the RGD sequence. Histological and histomorphometric examinations after 4 and 8 weeks showed a significant increase in bone growth in the RGD-Ti group compared to the control group.

BMPs belong to the transforming growth factor beta (TGF-β) family and are biological factors with a strong ability to induce bone, cartilage and connective tissue formation through the differentiation of bone mesenchymal stem cells (Dolanmaz et al., 2015b). They have been investigated as one of the growth factors (GF) that stimulate undifferentiated cells to become osteoblasts, with a certain ability to attract undifferentiated mesenchymal cells, regulating angiogenesis, chemotaxis and cell multiplication (Chang et al., 2010; Öncü and Alaaddinoğlu, 2015; Öncü et al., 2016). Numerous studies have reported that the use of BMPs improves the process of osteogenesis, osteoblast activity and osseointegration after dental implantation (Chen et al., 2004; Halloran et al., 2020). Nine of the reviewed studies (Wikesjö et al., 2008a; Wikesjö et al., 2008b; Wikesjö et al., 2008c; Ishibe et al., 2009; Ramazanoglu et al., 2011; Jiang et al., 2013; Kim et al., 2015; Yoo et al., 2015; Pang et al., 2021) used BMP-2 as a Ti implant coating. Wikesjö et al. used recombinant human bone morphogenetic protein-2 (rhBMP-2) in three studies and in different experimental models (Wikesjö et al., 2008a; Wikesjö et al., 2008b; Wikesjö et al., 2008c); in one study with non-human primates (Wikesjö et al., 2008c), they found that Ti surface coated with rhBMP-2 accelerated type IV bone formation; another study, in a canine model (Wikesjö et al., 2008b), based peri-implant bone remodeling on rhBMP-2 doses, reporting that sites receiving implants coated with rhBMP-2 at 3 mg/ml, showed increased formation of immature trabecular bone. On the contrary, the same authors in a third study, also on a canine model (Wikesjö et al., 2008a), demonstrated that rh BMP-2 at doses of 0.75 or 1.5 mg/ml, despite inducing osseointegration, did not increase BIC values, resulting significantly higher in the control group (uncoated Ti). Similarly, Ramazanoglu et al. (Ramazanoglu et al., 2011) found no increase in BIC in Ti implants with rhBMP-2 biomimetic coatings, despite inducing an improvement in peri-implant bone density.

Anitua et al. (Anitua et al., 2007; Anitua et al., 2009) proposed implant wetting with autologous growth factors, obtaining significant improvements in osseointegration. Lee et al. (Lee et al., 2010) reported that Ti porous oxide implants coated with rhBMP-2 significantly induce bone formation and remodeling, although they did not find significant effects according to the application techniques.

The ECM is a three-dimensional network, with an abundance of macromolecules, such as type I collagen, proteoglycans, laminin and fibronectin, which provides biochemical and structural support to surrounding cells (Daley and Yamada, 2013). It has been highlighted that ECM could affect the differentiation, survival and potentiality of mesenchymal stem cells (MSCs) by modulating the activity of growth factors and affecting cell behavior (Assis-Ribas et al., 2018). Feng et al. in a recent investigation (Feng et al., 2020) studied the behavior of MSC laminates, obtained by a decellularization process, on SLA-surfaced implants and demonstrated that they promoted adhesion, proliferation and osteogenic differentiation of bone marrow mesenchymal stem cells (BMSCs) *in vitro*, and improved osseointegration of implants *in vivo*. Shekaran and Garcia in a review study (Shekaran and García, 2011) highlighted the functionalization of implants with ECM peptides or proteins, to modulate host cell responses to the implant material and to enhance osseointegration and bone formation. They also observed that surfaces presenting the peptide Gly-Phe-Hyp-Gly-Glu-Arg (GFOGER), from the α1 chain of type I collagen, promote osteoblastic differentiation of primary bone marrow cells *in vitro*, and that GFOGER-functionalized titanium implants would improve implant integration in a rat cortical model by enhancing peri-implant bone formation and implant attachment to bone. Despite this, studies such as those by Hennessy et al. (Hennessy et al., 2009) disagree with these results, suggesting that collagen mimetic peptides would exclusively stimulate osteoblastic differentiation and that the beneficial effects would be due to the role of these peptides as differentiation rather than adhesion factors. Stadlinger et al. (Stadlinger et al., 2008; Stadlinger et al., 2012b) in two *in vivo* studies did not obtain variations in BIC at 4 and 8 weeks after cycloaddition in collagen-coated implants, finding only a slight increase in bone-to-implant contact around the implants that incorporated CS in the coating and observing that the additional inclusion of a low amount of rhBMP-4 had a detrimental 4meta-analysis had several limitations: first, different experimental models were used, suggesting different bone formation dynamics, especially in early healing times (Pearce et al., 2007; Wancket, 2015). These factors may influence the observed BIC values. Second, this meta-analysis focused only on three biomolecular coatings (peptides, BMPs, and ECMs), leaving out other bioactive coatings; moreover, the coatings in the different studies were not single coatings, but most resorted to combined coatings. Thirdly, the discrepant follow-up periods (2–84 weeks) and differences in the number of animals in the studies, could condition the results. Fourth, the various investigations analyzed several parameters indicative of bone neoformation and in our meta-analysis only BIC was chosen as a measure indicative of osseointegration (Albrektsson and Johansson, 2001; Gehrke et al., 2020).

## 5 Conclusion

In summary, the present meta-analysis revealed that the use of certain bioactive organic molecules seems to promote peri-implant bone formation, which could influence osseointegration during the early stages of healing; however, different factors make comparison between studies difficult and complicate the interpretation of the results on peri-implant bone formation. Nevertheless, in order to confirm the clinical applicability of these findings, in addition to a greater number of preclinical studies on suitable experimental models, clinical trials with prolonged follow-up periods would be necessary, since the results of preclinical experiments do not necessarily reflect the human clinical reality.

## Data Availability

The original contributions presented in the study are included in the article/Supplementary Material, further inquiries can be directed to the corresponding author.
